# Coexistence of Pemphigus Vulgaris and Lichen Planus following COVID-19 Vaccination

**DOI:** 10.1155/2022/2324212

**Published:** 2022-08-28

**Authors:** Zeinab Aryanian, Kamran Balighi, Arghavan Azizpour, Kambiz Kamyab Hesari, Parvaneh Hatami

**Affiliations:** ^1^Autoimmune Bullous Diseases Research Center, Tehran University of Medical Sciences, Tehran, Iran; ^2^Department of Dermatology, Babol University of Medical Sciences, Babol, Iran; ^3^Department of Dermatology, Razi Hospital, Tehran University of Medical Sciences, Tehran, Iran; ^4^Department of Dermatopathology, Razi Hospital, Tehran University of Medical Sciences, Tehran, Iran

## Abstract

SARS-CoV-2 vaccines were approved without long-term monitoring due to emergent situation and might have several side effects. Herein, we describe the first case with development of both LP and PV following COVID-19 vaccination. Immunological alteration due to COVID-19 vaccination and its potential role in triggering autoimmune disorders were also dealt with.

## 1. Introduction

Coronavirus disease 2019 or COVID-19 caused by SARS-CoV-2 was first reported in December 2019 and soon became a pandemic in March 2020 [1]. Since serious health issues were raised regarding COVID-19 outbreak, SARS-CoV-2 vaccines were approved without long-term monitoring and it turned out that they can cause several adverse effects including mucocutaneous manifestations [2–5].

tLichen planus (LP) is a chronic inflammatory dermatosis, involving skin, appendages, and mucous membranes [6, 7].

Pemphigus vulgaris (PV) is a rare blistering disorder with mucocutaneous involvement characterized by the production of autoantibodies against desmogleins (Dsg), which play an important role in the maintenance of cell-to-cell adhesion [8].

There are some reports of development of lichen planus-like lesions in pemphigus patients [9–11].

Although the presence of LP or PV following COVID-19 or its vaccines has been rarely reported, their coexistence following COVID-19 vaccination has not been reported yet.

Here, we present the first case of coexistence of LP and PV following COVID-19 vaccination.

## 2. Case Presentation

A 43-year-old man was referred to our clinic for evaluation of mouth sores and dark spots on the face from three months ago. He was otherwise healthy and had no notable drug history. His initial symptom was dysphonia which developed quite abruptly within 48 hours of receiving the second dose of the Oxford-AstraZeneca COVID-19 vaccine, though he did not report any adverse effect following receiving the first dose of the same vaccine. He had progressed to develop sore throat and erosive buccal lesions. Curiously, he also noted increasing dark spots on his face at this time which were not proceeded by blisters or ulcers. Histopathologic sampling and direct immune fluorescence evaluation of a dorsal tongue ulcer by an outside dermatologist confirmed the diagnosis of pemphigus vulgaris (PV). Treatment had been initiated with 80 mg/day prednisolone and 150 mg/day azathioprine with partial control of the disease. The patient was subsequently referred to our clinic for further management.

**Figure 1:**
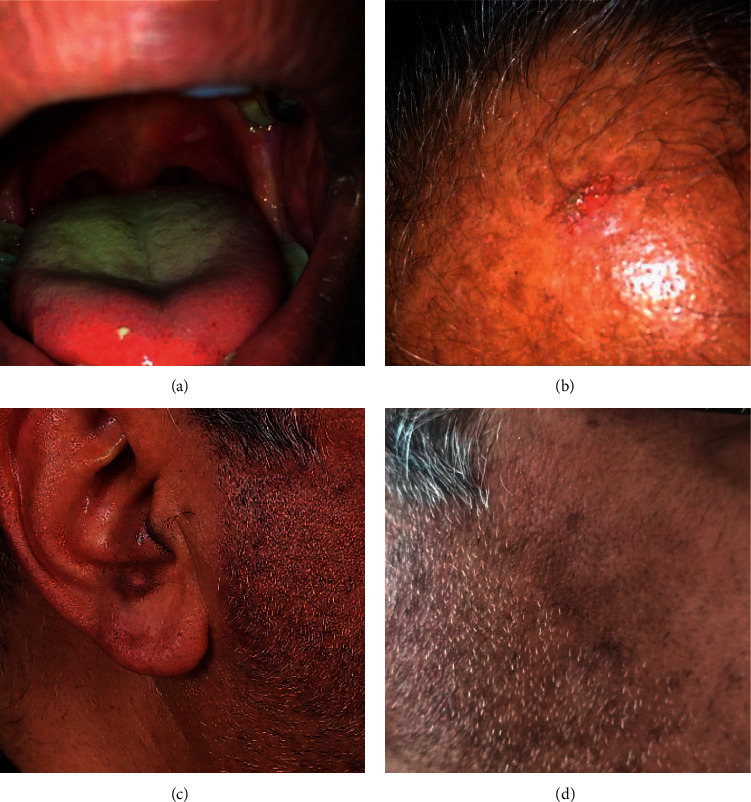
Oral mucosal lesions (a) and scalp erosion (b) due to pemphigus and hyperpigmented macules on ear (c) and cheek (d) due to lichen planus pigmentosus following COVID-19 vaccination.

**Figure 2:**
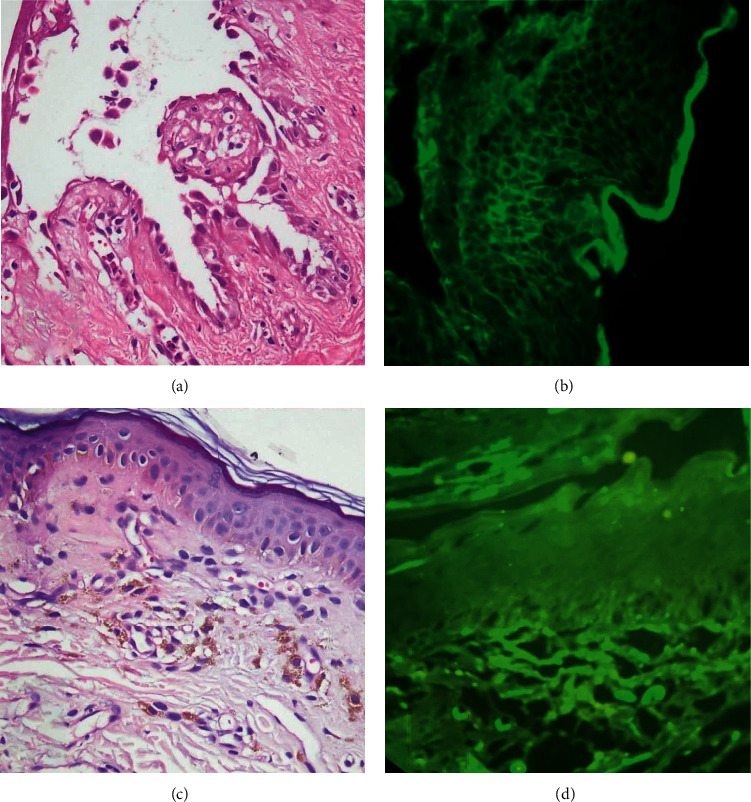
Suprabasal epidermal acantholysis and cleft containing inflammatory cells and rounded acantholytic cells with intensely eosinophilic cytoplasm and a perinuclear halo. DIF shows intercellular deposition throughout the epidermis compatible with diagnosis of PV (a, b). Hyperkeratosis, atrophic epidermis with vacuolar alteration of the basal cell layer, and pigmentary incontinence and negative. DIF, compatible with diagnosis of LP pigmentosus (c, d)

Upon examination, residual PV erosions were found on oral palatal mucosa and scalp (Figures 1(a) and 1(b)). We also noticed multiple well-defined grey-brown hyperpigmented macules on the ears, cheeks, and chin (Figures 1(c) and 1(d)). He denied exposure to any topical medications or potential for personal care product/occupational contact dermatitis. Since the nature of this pigmentation was unclear, we performed a punch biopsy of a representative chin lesion which showed typical findings of lichen planus (LP) pigmentosus (Figure 2).

We started a topical corticosteroid (mometasone cream qhs) for the LP lesions with recommendation for diligent sun protection. Considering the incomplete response of PV to azathioprine, rituximab was initiated (500 mg every week for 4 sessions) which resulted in considerable clinical response and tapering the prednisolone. At present, his old PV lesions have been healed without developing any new lesion on 5 mg/day prednisolone and his LP lesions, although partially, have been improved.

**Figure 3:**
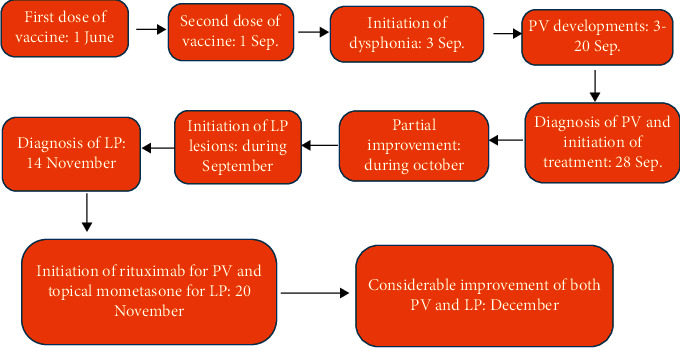
The exact time points of events.

Figure 3 shows the exact time points regarding the history of the patient. It is worthy to note that patient did not have any positive personal or family history regarding other immunological disorders such as rheumatoid arthritis, type 1 diabetes, autoimmune thyroid disease, irritable bowel disease, and systemic lupus erythematosus. Moreover, no one in his family showed any immunologic reaction following COVID-19 vaccination.

## 3. Discussion

Coexistence of LP and PV has rarely been reported. Most previously reported cases had the oral forms of both diseases [9–11]. PV and LP share some immune pathogenic features including aberrant immune responses. LPP is mainly derived by cell-mediated immunity while the major mechanism of developing PV is auto antibody formation against desmoglein (Dsg) 1, 3 [12, 13]. T cells, however, are implicated in the pathogenesis of PV. Furthermore, circulating anti-Dsg 1, 3 in some patients with oral LP has been reported, suggesting possible shared immunogenic pathways [13].

Whenever a mixed picture of PV is concurrent with LP-like features, one should consider the critical paraneoplastic pemphigus [14]. However, our patient did not have severe refractory stomatitis which is the clinical hallmark of paraneoplastic pemphigus. The histopathology and DIF pattern were also not suggestive of paraneoplastic pemphigus.

The new-onset occurrence of LP or PV, individually, has been reported following COVID-19 infection or various COVID-19 vaccine platforms including AstraZeneca or Pfizer, which was presented both after first or second dose of vaccine [15–17]. This might reflect that the dose of vaccine is not of a great importance in triggering autoimmune condition.

The simultaneous occurrence of cutaneous LP pigmentosus and PV triggered by COVID-19 vaccination in our case, to the best of our knowledge, has not been previously reported in the literature.

It has been long known that autoimmune disorders can be triggered by various environmental factors such as viral infections or vaccinations in patients with permissible genetic backgrounds. COVID-19 vaccines can elicit a T-helper type 1(Th1) response, increasing pro-apoptotic mediators such as interferon gamma (IFN *γ*), thereby explaining occurrence of LP following vaccination [18]. Though PV is supposed to be a Th2-dominant disease, some studies suggested a possible role of CD8+ T cells via Fas/Fas ligand pathway in the pathogenesis of PV [19]. On the other hand, COVID-19 vaccines have been shown to have a high capability to elicit critical immune regulators like cytotoxic CD8+ T cells and memory cells [20] which might explain the occurrence of PV in this setting. Moreover, COVID-19 vaccines, especially adenovirus-containing ones, might render an environmental trigger for induction of autoimmune disorders such as PV in genetically susceptible patients possibly through epitope spreading or bystander activation phenomenon [21].

Although it seems that the pandemic is ending, COVID-19 vaccination will continue in future. Hence, it is needed to know more about COVID-19 vaccine-associated dermatologic reactions. Logically among them, we may encounter some rare but important and even life-threatening reactions that should be better studied and managed. We recommend a strict workup and report of development of new autoimmune disorders and unusual dermatologic conditions to better understand immune system work frame and early treatment of disorders (but not too early which interfere with immunologic body response to vaccination).

## Data Availability

The data that support the findings of this study are available from the corresponding author upon reasonable request.
